# Radiation Risk in 2D Mammography Screening: A Scoping Review of Modelling Strategies and Emerging AI Applications

**DOI:** 10.1002/jmrs.70022

**Published:** 2025-10-03

**Authors:** Nazli A. Moda, Mo'ayyad E. Suleiman, Sahand Hooshmand, Warren M. Reed

**Affiliations:** ^1^ Faculty of Medicine and Health, Discipline of Medical Imaging Sciences The University of Sydney, Susan Wakil Health Building (D18) Sydney Australia

## Abstract

Breast cancer is the most commonly diagnosed cancer among women worldwide, and concerns regarding radiation exposure from mammography screening remain a potential barrier to participation. This scoping review explores existing models estimating long‐term radiation risks associated with repeated mammography screening. A structured search across five databases (Medline, Embase, Scopus, Web of Science and CINAHL) along with manual searching identified 24 studies published between 2014 and 2024. These were categorised into three themes: (1) models estimating dose–risk profiles, (2) factors affecting radiation dose and (3) the use of artificial intelligence (AI) in dose estimation and mammographic breast density (MBD) estimation. Studies showed that breast density, compressed breast thickness (CBT) and technical imaging parameters significantly influence mean glandular dose (MGD). Modelling studies highlighted the low risk of radiation‐induced cancer, inconsistencies in protocols and vendor‐specific limitations. AI applications are emerging as promising tools for improving individualised dose–risk assessments but require further development for compatibility across different imaging platforms.

## Graphical abstract

This scoping review examined models estimating long‐term radiation risks from repeated mammography screening. While modelling indicates the risks are low, approaches vary by protocol and vendor. Emerging AI tools show promise for improving personalised risk estimation, though further development is needed for cross‐platform compatibility.

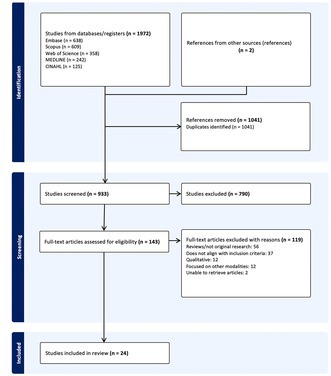

## 1 Introduction

Breast cancer is the most frequently diagnosed cancer among women worldwide, accounting for 23.8% of new cancers and a 12.7% mortality rate in 2022 [1]. The age‐standardised incidence rate in the United States (U.S.) and Australia was 95.9 and 101.5 per 100,000, respectively [2]. Breast cancer cases represent 15.5% of all new female cancer cases in the U.S. [3] and 28% in Australia [4]. Mammography has been identified by the International Agency for Research on Cancer (IARC) as the most effective tool for reducing breast cancer mortality in women aged 50–69 [5]. Small breast cancers, defined as less than or equal to 15 mm by BreastScreen Australia, are linked to greater treatment options, increased survival and decreased morbidity [6]. In 2022, 58% of breast cancers detected through BreastScreen Australia in women aged 50–74 were classified as small (≤ 15 mm) [6], underscoring the importance of early detection through mammography screening.

Since its inception in 1991, BreastScreen Australia has contributed to a decrease in breast cancer mortality from 74 to approximately 37 deaths per 100,000 women in 2022 [6]. The 5‐year relative survival rate for Australian women aged 50–74 rose from 73.8% (1986–1990) to 94.1% (2016–2020) [6]. In the U.S., the Surveillance, Epidemiology and End Results [7] (SEER) data reported a 5‐year relative survival rate of 91.7% (2015–2021) [3]. Despite these benefits, participation rates in BreastScreen Australia remain around 50% of the eligible population [6]. Barriers include limited awareness, concerns over radiation exposure, breast cancer diagnosis and treatment [8, 9]. Debate around the lifetime risk of radiation‐induced cancer from screening has persisted for decades [10]. Screening interval and commencement age guidelines differ by country, affecting cumulative radiation dose and associated risk. Australia recommends biennial screening for women aged 50–74 [11]; the United Kingdom (U.K.) offers triennial screening for ages 50–71 [12]; and the U.S. recommends biennial screening for ages 40–74 [13].

Public concern about radiation levels remains high; therefore, clear and transparent information is required about radiation levels in medical imaging, such as the comparative dose per examination. The Australian Radiation Protection and Nuclear Safety Agency (ARPANSA) states that 5000 millisieverts (mSv) can be lethal, with cancer risk increasing at 100 mSv or above and potential health effects even at lower levels [14]. Australians receive approximately 1.7 mSv of background radiation annually from environmental sources [14]. The integration of artificial intelligence (AI) into breast imaging is a more recent development, particularly in areas such as image interpretation, breast cancer prediction and, in some studies, dose estimation [15, 16, 17]. AI also enables more accurate measurement of mammographic breast density (MBD) [18, 19], a key factor influencing mean glandular dose (MGD) calculations.

This scoping review explored current modelling approaches used to estimate the lifetime radiation risk from mammography screening. Given the variability in imaging platforms, a consistent and standardised method for dose–risk evaluation is essential. Radiation risk modelling enables personalised risk assessments by accounting for changes in breast tissue composition and compressed breast thickness (CBT) over time. Therefore, the aim of this review was to evaluate existing dose–risk models used in mammography screening, assessing their limitations and highlighting areas for improvement. Ultimately, the findings are intended to support radiographers, patients and policymakers in making better informed decisions about breast cancer screening.

## 2 Materials and Methods

This scoping review followed the Preferred Reporting Items for Systematic Reviews and Meta‐Analysis extension for Scoping Reviews (PRISMA‐ScR) Checklist [20]. A scoping review was selected to map wide‐ranging evidence, explore existing concepts and identify the current gaps in the literature across modelling and AI applications.

### 2.1 Eligibility Criteria

Studies were included if they met the following:

1. Original research published in English in peer‐reviewed journals between January 1, 2014, and May 13, 2024.
2. Investigated radiation dose or associated risk profiles in standard 2D mammography.
3. Examined factors influencing MGD, or included models or methods to estimate or categorise individual dose profiles.

Studies were excluded if they were:

1. Focused on other imaging modalities other than standard 2D mammography, such as magnetic resonance imaging (MRI), digital breast tomosynthesis (DBT) or ultrasound.
2. Were editorials, conference papers, commentaries, case reports or review articles.

This review aimed to focus on original empirical studies providing quantitative data on dose estimation or risk modelling in standard 2D mammography. Review articles, editorials and other non‐empirical formats were excluded to ensure consistency in methodological quality. The 10‐year date range reflects contemporary practice, coinciding with the increasing use of digital mammography and emerging AI tools in dose estimation.

### 2.2 Information Sources and Search Strategy

A structured literature search was conducted using five databases: Medline, Embase, Scopus, Web of Science and Cinahl. The search strategy used the keywords: (mammogram* AND screening) AND (“breast cancer”) AND (radiation AND risk) OR (radiation AND dose). A manual search of reference lists from relevant articles was performed to identify any additional relevant studies.

### 2.3 Study Selection

One author/reviewer (N.A.M.) conducted the initial search. Title and abstract screening was independently completed by two author/reviewers (N.A.M. and S.H.), with any discrepancies resolved by a third author/reviewer (M.E.S.). Full‐text screening for eligibility was performed by two authors (N.A.M. and M.E.S.), following the predefined inclusion and exclusion criteria.

### 2.4 Data Charting

A customised data charting form was developed to extract key information from each included study. The form was piloted and refined in consultation with the review team before full data extraction. Thematic analysis was used to categorise studies into major themes. Tables 1, 2, 3 in the Results section were structured according to relevant groups derived from the literature, namely dose models, radiation dose factors and AI applications.

## 3 Results

### 3.1 Study Selection

**FIGURE 1:**
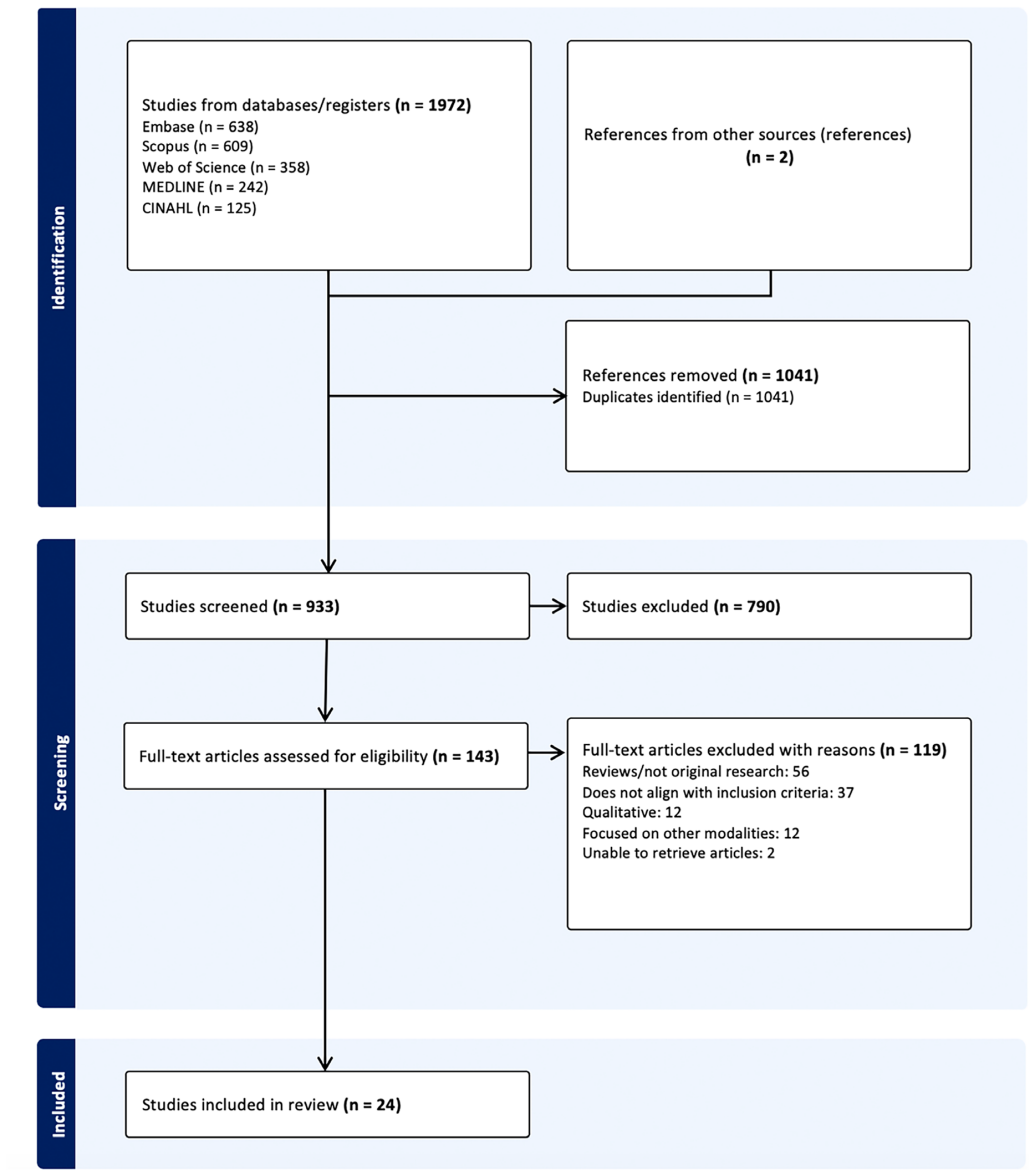
PRISMA flow chart of study selection process.

A total of 1972 articles were identified through the initial database search. After removing 1041 duplicates using Covidence, 933 articles remained for title and abstract screening. Of these, 790 were excluded, and 143 articles underwent full‐text review. Following eligibility assessment, 24 studies were included in the final review. Two additional articles were identified through hand‐searching reference list screening. The PRISMA flow chart illustrating the study selection process is provided in Figure 1. Of the included studies, 11 focused on modelling, 9 on factors affecting radiation dose and 4 on AI applications.

The subsequent thematic analysis summarised in Tables 1, 2, 3 supports the findings described below, enabling identification and comparison of modelling studies, screening protocols, and associated radiation dose risk. Together, these tables highlight the effectiveness of dose models, the key factors influencing MGD, and the emerging potential of AI in mammography screening [16, 17, 18, 19, 21, 22, 23, 24, 25, 26, 27, 28, 29, 30, 31, 32, 33, 34, 35, 36, 37, 38, 39, 40].

### 3.2 Radiation Dose and Risk Estimation

Quantifying radiation dose and associated risks in medical imaging is essential for understanding the potential carcinogenic effects of radiation exposure. Several studies used frameworks, such as the BEIR VII Phase 2 report, to quantify lifetime attributable risk (LAR) of radiation‐induced cancer [41]. LAR accounts for several factors including dose, age and sex and provides conversion factors to estimate incidence and mortality for body sites for a population of 100,000 exposed to 0.1 gray (Gy) [41]. The linear no‐threshold (LNT) model linearly extrapolates carcinogenic risks using known data of high radiation dose impacts to determine effects of low doses, and thus it has been criticised for possible over‐ or underestimation of risk at low doses, as it assumes that any level of radiation exposure carries risk [41, 42]. The dose and dose–rate effectiveness factor (DDREF) acts as a conversion factor for the LNT model utilising existing epidemiological data and nuclear events to translate the known impacts of these high doses and high‐dose rates to determine risk for low doses and low‐dose rate exposures used across medical imaging [41, 43]. DDREF value recommendations exist in the literature, for instance, the ICRP 2007 recommends a DDREF of 2 [43] while the BIER VII Phase 2 report suggests a DDREF of 1.5 [41].

MGD is the preferred metric for quantifying radiation dose in breast imaging. Breast tissue is radiosensitive, with a tissue weighting factor of 0.12 [43, 44]. For context, an average CT scan delivers around 10 mSv, a chest X‐ray is 0.1 mSv [45], and a mammogram typically involves 1.43 milligray (mGy) for mediolateral oblique (MLO) projections and 1.36 mGy for craniocaudal (CC) projections [32]. A typical mammography examination delivers an effective dose of approximately 0.33 mSv, which is about three times higher than a standard chest X‐ray, yet significantly lower than the dose from a typical CT scan (Tables 1, 2, 3).

**TABLE 1:** Mammography dose models.

References	Aim	Methods	Key findings
[21]	Investigate the radiation‐induced cases, death and lives saved from mammography screening	Published dose data, calculations for 100,000 females, aged 50–69, biennial screening, follow‐up till 85 or 105 years	50–85 years, 2.5 mGy, latency time of 10 years, DDREF of 1 Lifetime risk of radiation‐induced breast cancers per 100,000 females = 10 (95% CI: 4–25) Radiation‐induced breast cancer death = 1 (95% CI: 0–2) Assumed number of lives saved = approximately 350
[22]	Establish a method for effective lifetime risk of radiation‐induced cancer from mammography screening	280 dosimeters used in a dosimetry phantom to calculate dose Organ dose measured and MGD estimation	Average risk (47–73 years, total 9 screens) = 93 case per million High risk (40–73 years, total 34 screens) = 489 case per million
[23]	Develop a method to measure glandularity using pixel values in mammograms	314 MLO mammograms Glandularity quantification and radiation dose	Assumed glandularity overestimates smaller breasts MGD
[24]	Estimate the radiation risk of induced cancer and mortality from mammography, including diagnostic images	Microsimulation of Screening Analysis‐Fatal Diameter model Simulation model of radiation exposure	Annual screening, 100,000 women (40–74 years) = 125 breast cancer radiation‐induced cases (95% CI: 88–178), 16 deaths (CI: 11–23). 968 breast cancers avoided
[25]	Investigating the number of radiation‐induced cancers versus the benefits and lives saved from breast screening in England	Assumption of 1,770,436 females undertaking mammography screening between 50‐70 years to determine the ratios	Lives saved from screening to radiation‐induced cancer mortality ratio: DDREF of 1 = 156:1 DDREF of 2 = 312:1 1.8% with very thick breasts, the ratios decrease to DDREF of 1 = 94:1; DDREF of 2 = 187:1
[26]	Modelled lifetime radiation risk from FFDM screening (for average breast size and MBD)	2 breast phantoms used on 16 FFDM machines Effective risk calculations. Regression model	Risk impacted by commencement age. In a population of 100,000: 30 years = 5.19 cases; 40 years = 2.9 cases; 50 years = 1.44 cases
[27]	Develop a model with physical factors to predict glandulartiy and radiation dose before screening	331 MLO views Pixel values in adipose‐only and glandular areas identified. Glandularity and MGD evaluated	MGD = 1.322 mGy Significant prediction MGD regression model; *p* < 0.0001
[28]	Establish a risk model that evaluates an individual woman's risk of radiation‐induced breast cancer from one mammography screening visit	31,097 images Retrospective analysis and modelling	Medium risk female attending biennial screening 50–74 years = 29.6 radiation‐induced breast cancer cases per 100,000 females
[29]	Extension of Breast‐iRRISC to calculate dose and risk profile, lifetime MGD for screening ages of 40–75 years	20,232 images MGD predictions for all screening examinations Dose category established with lifetime effective risk calculated	Biennial screening, 50–74 years incidence and mortality risk per 100,000 females Low dose category = 8.64 and 2.61 Medium dose category = 11.76 and 3.55 High dose category = 15.08 and 4.55
[30]	Evaluate the RRIMS model by comparing the predictions from the model to manually calculated dose to ensure consistency and accuracy	2930 images RRIMS predictions compared and analysed with manual calculations	Intraclass correlation coefficient (ICC, 3,1) = 0.64 (*p* < 0.001) Kendall's W = 0.83 (*p* < 0.001)
[31]	Determine impacts of mammography screening protocols in Brazil	Simulation study conducted utilising available breast cancer screening incidence and mortality data	Biennial screening 60–69 age category was the most optimal: net benefit of screening = 31.159

**TABLE 2:** Factors affecting radiation dose.

References	Aim	Methods	Key findings
[32]	Investigating radiation doses in Australia during mammography screening using CBT as an indicator for DRLs	52,405 mammograms Retrospective analysis Dose calculation and DRLs calculated—75th, 95th percentiles calculated for the median image MGDs for each mammography unit	Median image MGD: MLO = 1.43, CC = 1.36 mGy Individual doses per image: MLO = 0.32 to 10.00 mGy, CC = 0.19 to 7.45 mGy Median MGD per examination = 2.84 mGy
[33]	Investigate the impact of mammography radiation doses on DNA	Total of 18 specimens Irradiations replicated low dose mammography	RBE = 1.82 (low‐dose range) RBE = approximately 1 (high‐dose range) Low dose hypersensitive response for double‐stranded breaks which is applicable to mammography doses
[34]	Estimates radiation risk of FFDM screening programmes nationally	Phantom study 16 FFDM machines Effective lifetime risk calculated for 48 screening programmes	Significant differences observed between countries: Malta = 42 cases/10^6^, U.S. = 194 cases/10^6^
[35]	Assess the carcinogenic risk of mammography screening	427 women Views—CC, MLO Breast density categorised Radiation dose and risk estimations	MLO MGD higher than CC Mean dose per projection: MLO = 2.63 mGy, CC = 2.12 mGy
[36]	Examines the biological effects of low doses used in mammography	Three epithelial cell lines used Irradiations replicated MGD	Increases apoptosis and DNA double‐strand breaks
[37]	Investigates the impact of a family history on breast cancer risk from radiation exposure	17,200 women (1079 breast cancer cases between 1958 and 2013) Excess absolute risk model utilised to determine familial risk due to radiation	Risk increases by 2.7 times if a first‐degree family member (mother, sister, daughter) was previously diagnosed with breast cancer
[38]	Investigates the radiation risk of mammography screening	126 projections Views—CC, MLO MGD calculated, risk estimations evaluated	LAR per 100,000 women: Mammogram = 1.64, MLO = 0.88, CC = 0.76
[39]	Investigates radiation dose received in mammography screening	510 mammograms Views—CC, MLO Radiation dose and risk calculations performed	Connection between exposure parameters, breast thickness and MGD (*p* < 0.01)
[40]	Investigated MGD for CC and MLO by entrance skin dose. Regression analysis of patient age, acquisition parameters and breast thickness	2035 mammograms MGD estimation and regression analysis	Mean and range MGD: CC = 0.832 (0.110–3.491) mGy MLO = 0.995 (0.256–2.949) mGy Lower MGD for 64 years and older

**TABLE 3:** AI applications.

References	Aim	Methods	Key findings
[19]	Create a deep learning framework for MBD estimations	2416 images Breast and dense area segmentation—fully convolutional network Compute breast percentage density from segmentation outcomes	Algorithms rho = 0.85 LIBRA rho = 0.69 Statistically significant result of algorithm compared with BI‐RAD (*p* = 0.0001 or less)
[18]	Determine the performance of an AI algorithm in estimating MBD measurements compared with traditional methods	15,661 images 3 AI algorithms: Removal of FFDM image background and pectoralis muscle (deep learning models) Segmentation of dense versus fatty tissue and MBD estimations (radiomic machine learning model)	Strong association between AI and established methods; Cumulus, LIBRA, Volpara, clinical BI‐RADS, *r* = 0.90, 0.76, 0.89, 0.80, respectively
[16]	Prior to exposure to x‐rays, identify ESAK by a neural network	224 samples Multilayer perceptron network used with back‐propagation learning method	4.3% variation between the kerma identified by dosimeter compared with the network estimation
[17]	Investigate a neural network to determine ESAK and thus MGD	224 samples Artificial neural network—Multilayer perceptron network used with hidden layers and multiple training algorithms	7.40% root mean square error, 0.91 *R* ^2^ (38 hidden layers)

Abbreviations: CBT, compressed breast thickness; CC, craniocaudal projections; DDREF, dose and dose–rate effectiveness factor; DRLs, diagnostic reference levels; ESAK, entrance surface air kerma; FFDM, full‐field digital mammography; MBD, mammographic breast density; MGD, mean glandular dose; MLO, mediolateral oblique projections; RBE, relative biological effectiveness.

### 3.3 Factors Affecting Radiation Dose in Mammography

#### 3.3.1 Patient Factors Affecting Radiation Dose

MBD decreases with age and women aged 64 and above generally receive lower doses due to lower glandularity [40]. Multivariate analysis revealed that increased MBD was associated with a 1.4‐fold increase in cancer risk, and menopausal status increased risk by 2.5 times [46]. Risk varied by individual factors such as age, MBD and genetic predisposition. For example, women with a family history of breast cancer have been shown to have an increased susceptibility to radiation‐induced breast cancer, with a 2.7‐fold increase in radiation‐related risk [37]. Women with larger breasts (CBT of 7.5 cm and over) typically require additional projections, which further increase dose [24, 47]. Patient tolerance during compression also contributes to dose variability [48].

#### 3.3.2 External Factors Affecting Radiation Dose

In Australia, the median MGD for mammography was 2.84 mGy, although this varied by manufacturer [32]. For example, Hologic systems produced higher doses, while Philips systems delivered the lowest [32]. MLO projections consistently resulted in higher doses than CC, likely a result of increased mAs requirements [35]. mAs demonstrated a correlation to MGD (*p* < 0.01) [39]. Radiographer technique during compression contributed to dose variations [48]. Studies reported that radiation exposure during mammography can induce biological effects, such as DNA damage and apoptosis, particularly in glandular breast tissue [36]. One study found a relative biological effectiveness (RBE) value of 1.82, indicating a hypersensitive DNA double‐strand break response at low doses [33]. Differences in screening protocols across countries contributed to variation in cumulative exposure and overall risk. For example, Malta's triennial screening programme for women aged 50–60 (4 screens) reported 42 cases per million total effective risk, while the U.S. biennial screening (ages 40–75, 18 screens) had rates of 194 cases per million [34]. For high‐risk women in the U.S., the estimated effective risk increased to 1100 cases per million [34]. Risk estimates for a typical four‐projection mammogram were a LAR of 1.64 per 100,000 women (0.88 for MLO, 0.76 for CC) [38]. A summary of these factors affecting radiation dose in mammography is presented in Table 4.

**TABLE 4:** Summary table of factors affecting radiation dose and risk.

Factor	Quantitative change in risk	Patient or external factor affecting radiation dose	Reference to studies
Screening interval and commencement age	Triennial vs. biennial screening. Increased cumulative exposure and radiation risk due to commencement age and screening intervals	External	Effective risk Malta (triennial—50–60): 42 cases/million U.S. (biennial—40‐75): 194 cases/million [34]
	Effective lifetime risk of radiation‐induced cancer by commencement age	External	Figures measured in a population of a 100,000: [26] 30 years = 5.19 cases, 40 years = 2.9 cases, 50 years = 1.44 cases
	Lower age—greater radiation dose received	Patient	Women 64 and over have lower glandularity—lower radiation dose [40]
Compressed breast thickness (CBT)	CBT influences MGD	Patient	CBT is an indicator for diagnostic reference levels [32] Patient tolerance for compression impacts dose [48]
		External	Radiographer technique for compression impacts dose [48]
Mammographic breast density (MBD)	Increased MBD—increased radiation dose	Patient	1.4‐fold increase in cancer risk (*p* = 0.014) [46]
Family history	Family history leads to increased susceptibility and risk of breast cancer	Patient	2.7‐fold increase in radiation risk (95% CI: 1.0, 4.8; *p* = 0.05) [37]
Additional projections	Larger breast size requires additional projections—increased dose False positives can require additional projections—increased dose	Patient	CBT ≥ 7.5cm [24] False positives—95th percentile—one fourth of dose received vs. one tenth at the mean [24]
Manufacturer variability	In Australia, MGD differed for manufactures	External	Hologic systems produced higher doses [32]. Philips systems delivered the lowest [32]
Acquisition parameters	MLO projections deliver higher doses than CC—increased mAs requirements [35]	External	mAs demonstrated a correlation to MGD (*p* < 0.01) [39]

### 3.4 Modelling Radiation Dose–Risk Profiles

Yaffe and Mainprize developed a foundational model estimating 86 radiation‐induced cancers and 11 deaths per 100,000 women following an annual screening regimen from ages 40 to 55, followed by biennial screening till 74 [49]. A Norwegian model based on biennial screening from 50 to 69 years predicted significantly lower risks: 10 radiation‐induced cancers and 1 death per 100,000 women [21].

Other regression models calculated lifetime cancer risk under different screening protocols: [26]

- Age 30: resulted in 5.19 cases/100,000
- Age 40: resulted in 2.9 cases/100,000
- Age 50: resulted in 1.44 cases/100,000

Miglioretti et al. [24] reported that annual screening from age 40 to 74 could result in 125 breast cancer cases and 16 deaths per 100,000 women, while preventing 968 breast cancer deaths, supporting a favourable risk–benefit ratio.

A Japanese research group developed a predictive model that used pixel values from digital mammograms to estimate breast glandularity and MGD [23]. The study revealed that manual measurements of glandularity limited scalability and tended to overestimate MGD [23]. Follow‐up studies improved model accuracy by incorporating additional variables such as age, body mass index and CBT [27]. The refined glandularity model demonstrated an *F*‐value of 58.3 (*p* < 0.0001), while the MGD prediction model showed a significant regression *F*‐value of 78.0 (*p* < 0.0001), both indicating a strong predictive performance [27].

Advanced modelling tools, including Breast‐iRRISC [28] and RRIMS [29], further stratified patients by MGD exposure levels, estimating breast cancer incidence and mortality across low, medium and high‐dose categories. RRIMS calculated the risk of radiation‐induced breast cancer incidence and mortality for biennial screening (50–74 years) per 100,000 women, using a DDREF of 2. The reported risks were [29]:

- Low dose: 8.64 cases/2.61 deaths
- Medium dose: 11.76 cases/3.55 deaths
- High dose: 15.08 cases/4.55 deaths

RRIMS predictions were validated against manual calculations, reinforcing its reliability [30]. However, the model was limited to Hologic imaging systems, highlighting the need for multivendor compatibility to ensure broader applicability. This lack of cross‐platform interoperability is a recurring limitation in many dose–risk models [22, 29]. These modelling studies collectively demonstrate the importance of screening commencement age and individualised dose modelling. They also demonstrate that, despite reduced mammographic sensitivity in dense breasts, early detection provides significant clinical benefits that outweigh the low radiation risk.

### 3.5 Quantifying Dose Across Screening Protocols

Ali et al. [22] used phantoms to assess organ specific radiation exposure during mammography, identifying exposures to adjacent tissues including the thyroid and bone marrow. Their study suggested that annual screening from ages 40 to 73 could lead to 489 radiation‐induced cancers per million, whereas triennial screening from ages 47 to 73 could reduce this to 93 per million [22]. A Brazilian study found that annual screening for women aged 40 to 49 nearly doubled the rate of false positives compared with biennial screening for ages 50–69 [31]. The net benefit of screening, measured as lives saved minus estimated radiation‐related fatalities in the model, was highest in women aged 60–69 [31]. An English study supported these findings, reporting a 30:1 benefit–risk ratio with a DDREF of 1 and 60:1 with a DDREF of 2 [25].

### 3.6 Artificial Intelligence Applications

Nabipour et al. [16, 17] explored the use of neural networks to estimate entrance surface air kerma (ESAK) and MGD using phantom‐based images. In 2021, their study reported that neural network‐based calculations showed only 4.3% variance compared to solid‐state dosimeter readings, highlighting the method's potential in pre‐exposure dose estimation [16]. However, the study did not account for factors such as tube output, which varies between mammography systems and may influence dose accuracy. A further enhanced version of their model achieved high correlation with measured values (*R* ^2^ = 0.91) and a low root mean square error (RMSE = 7.4%), supporting the feasibility of AI‐based dose prediction tools for mammography [17].

Traditional MBD classification using the BI‐RADS system is known to be subjective and dependent on the imaging vendor. AI tools, such as Deep‐LIBRA, offer more consistent and accurate assessments by removing background noise and non‐breast anatomical structures from mammographic images [18]. Cumulus and Volpara are established software tools used for automated breast density estimation [18]. Cumulus is a semi‐automated tool relying on visual thresholding, while Volpara uses volumetric analysis [18, 50]. These tools were used to evaluate and compare performance in the study [18]. In validation studies, Deep‐LIBRA showed strong correlation with established methods: Cumulus (*r* = 0.90), Volpara (*r* = 0.89), LIBRA (*r* = 0.76), BI‐RADS (*r* = 0.80) [18]. This demonstrates AI's ability to enhance analytical consistency and reduce the variability associated with human interpretation.

Breast size and tissue composition significantly affect MGD [24, 46, 47]. AI‐driven models for MBD estimation have the potential to assist with standardising dose–risk assessments. For example, Lee and Nishikawa [19] developed a deep learning model using 1208 CC and 1208 MLO images to automatically segment dense fibroglandular tissue. Their model showed strong correlation with BI‐RADS classifications (*ρ* = 0.85), whereas LIBRA displayed moderate correlation (ρ = 0.69) [19]. These findings support the potential of AI‐based tools for personalised, vendor‐neutral radiation dose calculation methods. Individualised and personalised in this review refer to tailoring estimates based on individual factors such as patient age, breast density and glandular composition. Vendor‐neutral refers to a calculation method that can be applied to all vendors.

## 4 Discussion

This scoping review analysed 24 studies investigating radiation dose and risk modelling in mammography screening, including contributing factors and emerging AI applications. Eleven studies focused on radiation dose–risk modelling, nine on patient and technical factors affecting MGD, and four explored AI applications in dose estimation and MBD assessment. The findings suggest that while modelling studies estimate carcinogenic risk from repeated mammography screening, there is considerable methodological variability. The results reaffirm that the benefits of mammography screening outweigh the associated carcinogenic risks. However, there is limited literature focusing on individualised and standardised approaches to radiation dose modelling.

Interpretation of these findings suggests that individual‐level models are preferable to population‐based models due to international differences in screening protocols that impact cumulative MGD and cancer risk [34]. However, most existing risk estimations are based on assumed average doses rather than individual characteristics. For example, Yaffe and Mainprize [49] assumed a MGD of 3.7 mGy, whereas Hauge et al. [21] used 2.5 mGy, and Warren et al. [25] used 3 mGy in their respective models. AI tools show potential to enhance model precision, especially through automated, accurate assessments of breast characteristics such as MBD [18, 19]. Notably, radiomic features used in AI‐based models have demonstrated effectiveness in producing consistent and precise MBD measurements [18]. However, current AI solutions are largely vendor‐specific, limiting their broader clinical implementation. These insights suggest a clinical need for the development of AI models that are compatible across imaging platforms to enable personalised risk assessments.

RRIMS [29], Warren et al. [25] and Lauby‐Secretan et al. [5] emphasise that the mortality benefits of mammography outweigh the relatively low risk of radiation‐induced cancer. For instance, RRIMS showed a 0.005% increase in mortality across 13 screenings for high‐dose groups [29], while annual screening of women aged 40 to 74 was estimated to prevent 968 deaths per 100,000 screened [24]. Similarly, an English model estimated 1071 lives saved annually among women aged 50–70 [25]. Risk modelling also showed a decrease in radiation‐induced cancer estimates from 51.9 per million at age 30 to 29 cases at 40 and 14.4 at age 50 [26]. These comparisons support this current review's conclusions that the benefit of mammography screening outweighs any associated radiation risk. Recent modelling efforts in the U.S., such as the TG 282 breast dosimetry model, aim to improve dose estimation by accounting for glandular tissue position [51]. This underscores the need to expand modelling research in the Australian context, considering its relevance for radiographers, policymakers and public health.

This review reiterates that breast composition varies between individuals and changes with age, which significantly affects MGD output. Differences in screening intervals, commencement ages and family history are contributing factors to carcinogenic risk. This review investigated current models and highlighted that a patient‐centred risk approach is required, focusing on factors such as a woman's age, MBD and CBT along with exposure parameters for personalised dose–risk assessments.

While AI offers opportunities for improved accuracy and individualised predictions, challenges such as data bias, regulatory constraints and integration into clinical practice remain. For instance, data bias could result in the model being trained on images that focus on a particular age group or mammography machine, inhibiting the usability and accuracy of the AI model. Practical implementation challenges include clinician acceptability and integration with existing reporting systems to improve workflow and efficiency. AI‐driven models could provide patient‐specific risk estimates before screening, supporting informed decision‐making and potentially improving public trust. Furthermore, these tools could serve as valuable educational resources for clinicians and policymakers, supporting more personalised and evidence‐based screening recommendations.

There are several limitations to this review. Some included studies had small sample sizes or relied on single‐view imaging, which may reduce statistical power and limit generalisability [23, 27]. The review also excluded DBT and focused only on literature from 2014 onwards, which may have omitted earlier foundational studies. DBT is a separate examination that utilises different acquisition methods and thus is not directly comparable to 2D mammography, which was the focus of this review. While many factors influence radiation dose in mammography, this review did not comprehensively examine all contributing variables as this was not its primary objective. Additionally, many reviewed models were vendor‐specific, limiting their applicability across different imaging platforms. Research into multivendor, AI‐driven dose estimation tools remains a key gap in the literature and warrants further investigation. Future research into an individual's breast characteristics and changes that occur over time could facilitate personalised dose–risk assessments.

## 5 Conclusion

This review synthesised the evidence on radiation dose and risk in 2D mammography screening, confirming that the benefits of screening outweigh the associated radiation risks. While modelling studies are useful in estimating lifetime MGD and cancer risk, their broader application is limited by vendor‐specific designs and a lack of patient‐specific modelling. AI has showed promising results in the estimation of breast density, with models capable of segmenting dense fibroglandular tissue more efficiently and accurately than traditional methods. Given the high prevalence of breast cancer, continued evaluation of screening models remains essential. Future research should prioritise the development and validation of AI‐driven, multivendor tools capable of personalised risk assessments. The next logical step is to design and validate such models within the Australian healthcare context, ensuring their applicability across diverse populations and imaging systems. Such tools can contribute to more tailored screening strategies and improved communication of risk, potentially enhancing patient understanding and confidence in mammographic screening.

## Disclosure

The authors have nothing to disclose.

## Conflicts of Interest

The authors declare no conflicts of interest.

## Data Availability

Data sharing not applicable to this article as no datasets were generated or analysed during the current study.
